# Robust sampling and preservation of DNA for microbial community profiling in field experiments

**DOI:** 10.1186/s13104-019-4187-2

**Published:** 2019-03-22

**Authors:** Anneloes E. Groenenboom, Eddy J. Smid, Sijmen E. Schoustra

**Affiliations:** 1 Laboratory of Genetics, Wageningen University and Research, Wageningen, The Netherlands; 2 Laboratory of Food Microbiology, Wageningen University and Research, Wageningen, The Netherlands; 3 Department of Food Science and Nutrition, School of Agricultural Sciences, University of Zambia, Lusaka, Zambia

**Keywords:** Filter paper disks, Field trial, Microbial community, Milk, Fermentation, DNA stabilisation

## Abstract

**Objective:**

Stabilising samples of microbial communities for DNA extraction without access to laboratory equipment can be a challenging task. In this paper we propose a method using filter paper disks for the preservation of DNA from diverse microbial communities which are found in a fermented milk product.

**Results:**

Small adaptations to the DNA extraction method used for liquid fermented milk delivered DNA of sufficient amounts and quality to be used for later analyses, e.g. full community 16S amplicon sequencing. The microbial community structure obtained via the filter paper method showed sufficient resemblance to the structure obtain via the traditional DNA extraction from the liquid milk sample. This method can therefore successfully be used to analyse diverse microbial communities from fermented milk products from remote areas.

**Electronic supplementary material:**

The online version of this article (10.1186/s13104-019-4187-2) contains supplementary material, which is available to authorized users.

## Introduction

Sequencing techniques are developing fast, resulting in more advanced and affordable ways to analyse DNA, such as characterising bacterial community structure using 16S amplicon sequencing. These high tech developments however are of no use if DNA samples are not well preserved before reaching the laboratory. The stabilisation of DNA is a challenge in field experiments where researchers do not have access to a laboratory with basic equipment, like a refrigerator and a sterile work environment. In some cases the DNA extraction and amplification can be performed in the field [1]. Often, biochemical solutions are being applied to stabilise the DNA [2–4]. More convenient for field trials would be the use of FTA paper [5, 6] or another solid carrier [7], as that increases the ease of storage and sending of the samples. Such low-tech methods deliver an important advantage for field researchers who want to stabilise DNA with minimal sample processing and a few materials. In this study we explored and validated the use of simple filter paper to stabilise bacterial DNA from a fermented dairy product without prior extraction of DNA. This method is compared to an often used method where liquid samples are quickly cooled after sampling and transported in cold conditions [8].

In the method we studied, a small drop of a traditional fermented milk product is transferred to the filter paper and left to dry. The dried paper including the drop can be stored for a long period of time at ambient temperature before the DNA extraction is performed. The subsequent DNA extraction method is a variation on the DNA extraction method used for liquid samples from fermented milk products. The dairy product used in this study, called Mabisi, is a traditionally fermented product found in rural areas in Zambia. As the fermentation in spontaneous, the bacterial community in the product is very diverse [9].

To study the composition of the microbial community in the fermented product, it is essential to quench microbial growth immediately after sampling. In a laboratory environment this would be done by cooling the sample to temperatures below 4 °C, which will slow down and stop the growth of the bacteria responsible for fermentation, but will keep them viable for later analyses. The filter paper method described here will also slow down and eventually stop bacterial growth, as it fixes the bacteria and dehydrates the medium. Although the bacteria will not be viable for analyses using culturing techniques, the DNA can be isolated from the papers and used for bacterial community analyses, such as 16S amplicon sequencing.

## Main text

### Methods

#### Preparing filter paper

Discs of filter paper (qualitative 413, 75 mm, VWR International, Radnor, United States) were placed in a Petri dish. Samples of 1 mL were spotted at the centre of the filter paper. The filter papers were left to dry at uncontrolled temperature in a hut without windows. Drying times varied between 30 min and 2 h depending on the thickness of the sample. Dried samples were stored in a Petri dish for up to 2 months before DNA extraction was performed.

#### Extraction of DNA

The DNA extraction method was adapted from Ercolini [10] and Schoustra [9]. To extract bacterial DNA from the milk environment, some steps are necessary to break down the casein structure. The high protein content in combination with the relatively high percentage of lipids makes the use of the other DNA methods less successful for milk samples. For DNA extraction from liquid samples, 1 mL of fermented milk was spun down (2 min, 12000 RPM), after which the supernatant was removed. The cells were re-suspended in a mix of 64 µL EDTA (0.5 M), 160 µL Nucleic Lysis Solution (Promega, Madison, United States), 5 µL RNAse (100 mg/mL), 120 µL lysozyme (10 mg/mL) and 40 µL pronase E (20 mg/mL). Samples were incubated for 60 min at 37 °C while being shaken at 350 RPM.

For the extraction of DNA from the filter paper discs, a piece, 2 by 2 cm, was cut from the middle of the filter paper with a sterile pair of scissors. This piece was positioned at the bottom of a 1.5 mL Eppendorf tube with a pair of tweezers. The cells were thoroughly suspended in a mix of 132 µL 0.5 M EDTA, 320 µL Nuclei Lysis Solution, 1 µL RNase, 240 µL lysozyme and 80 µL pronase E. Samples were incubated for 24 h at 37 °C and agitation of 350 RPM.

After the incubation step described above of both liquid samples and paper samples, a standard DNA extraction protocol was used, see Additional file 1: Supplement A.

#### Bacterial community reconstruction

For validation of the method in the laboratory the DNA of the extractions was amplified in the PCR and cloned into vectors to identify the DNA fragments in a clone library (see Additional file 1: Supplement B). The DNA extracts of the field samples, containing DNA from all organisms in the community, were sent for bacterial 16S rRNA gene amplicon paired-end sequencing of the V4 hypervariable region (341F-785R) on the MiSeq Illumina platform by LGC genomics (Berlin, Germany). For further data processing and statistics the QIIME pipeline [11], modified from Bik et al. [12] was used (see Additional file 1: Supplement C).

### Results

#### Validation of the method in the lab

Before testing the filter paper method in the field, the DNA extraction method was tested and optimised in the laboratory. Pure Mabisi samples and ten times diluted Mabisi samples were transferred on filter paper discs. The extracted DNA from these samples was compared to the DNA extracts from a undiluted liquid Mabisi sample. The DNA concentration in the paper with the undiluted and diluted Mabisi was 172.9 ± 0.3 ng/µL and 11.8 ± 1.6 ng/µL, respectively. The amount of DNA is comparable to the DNA obtained from the liquid sample 120.7 ± 16.5 ng/µL. Also the purity of the DNA extracts from the filter paper is comparable to that from the liquid DNA extracts as judged by the A260/A280 ratio (1.80 ± 0.05 and 1.83 ± 0.2, respectively).

The clone library of the DNA extracts was blasted against the NCBI database. The microbial communities of the two liquid samples and the two paper samples with diluted Mabisi contain similar species (Additional file 2: Figure S1). Variations can be found in both abundance, richness and type of species to which these reads blast. The sample size was not sufficient to make statistical inferences.

#### Validation of the method in the field

**Fig. 1:**
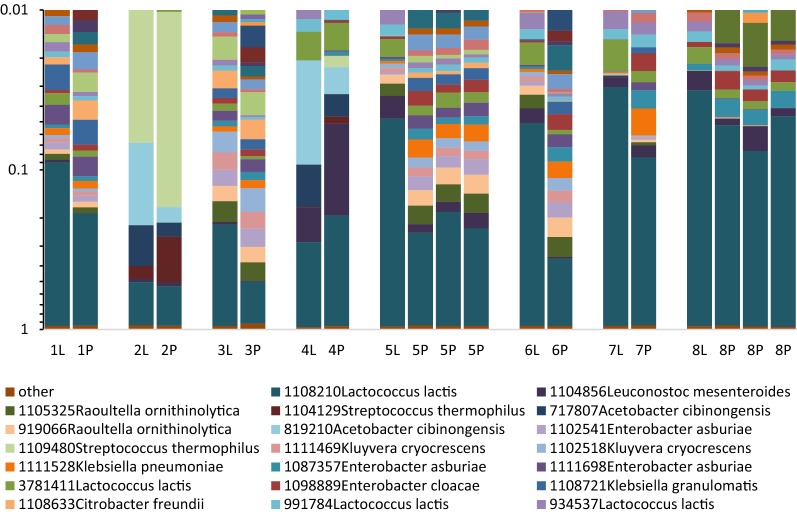
Microbial communities of paper samples and their corresponding liquid samples. Liquid samples are indicated with a L. Samples 5 and 8 have three technical replicates of DNA extraction from one filter paper disk. Different colours indicate different OTUs. BLAST results of the 20 most abundant OTUs are given

**Fig. 2:**
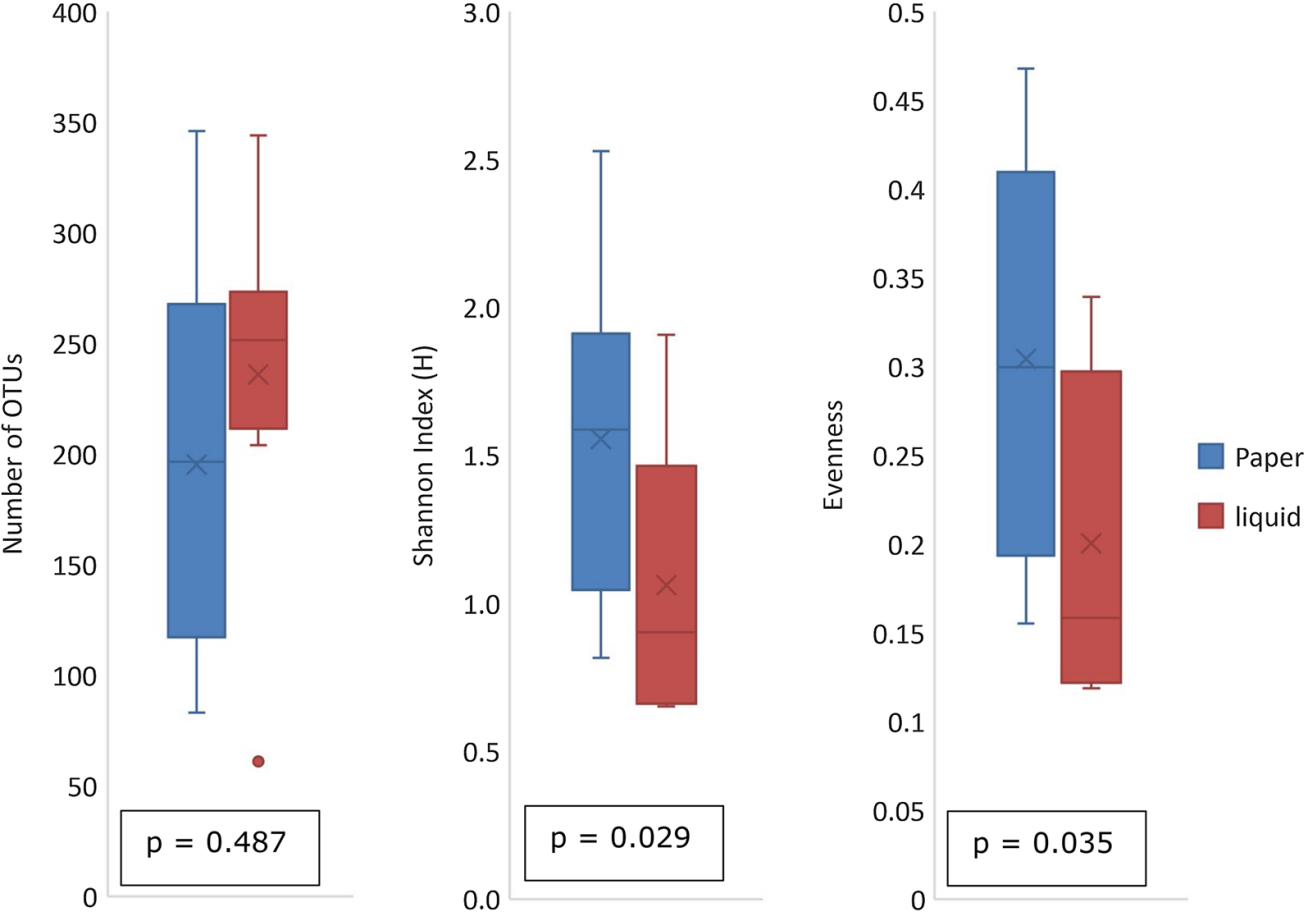
Number of OTUs, Shannon index and community evenness of filter paper samples and liquid samples. Comparison of eight samples which were all stored using the filter paper method as well as cooled to 4 °C before shipment from Zambia to the laboratory in the Netherlands were the DNA was extracted using the corresponding methods. P values are from the related-samples Wilcoxon signed rank test and show no difference between number of OTUs, but difference in OTU distribution (in both Shannon index and evenness)

At the field site in Zambia eight samples of Mabisi were split in two equal parts and subsequently transferred to filter paper as well as stored at 4 °C before shipment to our laboratory in the Netherlands. There the DNA was extracted using the corresponding methods. The concentration of DNA obtained from these extractions varied between 25 ng/µL and 125 ng/µL, with a higher average DNA concentration for the liquid samples (84.5 ± 25.1 ng/µL) compared to the filter paper samples (55.8 ± 35.4 ng/µL). To get similar concentrations of DNA for sequencing, the samples were diluted based on their DNA concentration to a concentration of 5–10 ng/µL. DNA extracts were sequenced (16S amplicon sequencing) using MiSeq Illumina sequencing and resulting reads were analysed to construct bacterial communities based on operational taxonomic units (OTUs). Comparison of the bacterial communities present in the samples shows a high degree of similarity between samples processed via the filter paper discs and those obtained by direct extraction of the fermentation liquid (Fig. 1). Most communities are dominated by *Lactococcus lactis*. In some samples also a high relative abundance of other bacterial species, belonging for example to the *Acetobacter* and *Streptococcus* genus, were found. In all cases, both collection methods (liquid and paper) show a similar microbial composition with differences in relative abundance. The differences in richness and diversity can be found in Fig. 2, where number of OTUs, Shannon index and evenness of all samples are plotted. This analyses shows no difference in total amount of OTUs that returned, but shows a difference in OTU distribution. Communities obtained via the paper method showed a higher Shannon index and a higher evenness.

### Discussion

The filter paper method described above can be used to determine the bacterial microbial communities in a fermented milk products produced in areas which have no access to basic laboratory equipment and cooling facilities. With some minor adjustments to the DNA extraction method for fermented milk, good quality DNA could be extracted from the filter paper disks. To a large extent this extraction from filter paper showed the same microbial community structure as the DNA extracted from the liquid sample directly. Although the same number of OTUs were obtained from the DNA extract the distribution was slightly different. The filter paper extraction seemed to have extracted relatively less of the most abundant OTU which represent *L. lactis* and relatively more of the OTUs present in low amounts. Filter paper is known to have bias towards preservation of certain species [13–15], and more research towards this will be necessary to validate this bias.

To analyse all aspects of fermentation it is essential to determine the bacterial community composition during, or even before, periods of vigorous growth. With our method, in which the bacterial growth is arrested due to drying of the milk, dynamic time course samples can be stabilised and subsequently analysed at a later stage. The samples analysed here are all from fermentation end points. Consequently, no active growing microbial cells are expected anymore in these samples due to the lack of nutrients and the low medium pH. Therefore, we can assume that at this particular stage, the bacterial community composition is already relatively stable. This makes it possible to compare DNA extractions from the filter paper with extraction from the liquid sample. Still the liquid sample cannot be seen as a perfect benchmark. Where the filter paper method might overestimate bacterial diversity, the DNA extracted from liquid sample might give an underestimation of the diversity. It is however a promising observation that the filter paper method was able to return low abundancy OTUs on top of identifying OTUs with high abundance. The method presented had proven its value and is sufficiently accurate for our purpose; the characterisation of the bacterial communities in a traditional fermented product. Besides, this method is easier and less expensive than the commercially available alternatives.

This method is also used for the characterisation of the bacterial communities in cereal based fermented foods from Benin, Zambia and Tanzania (unpublished observations, S. Phiri, S.E. Schoustra and A. Linnemann). Here the method also showed to be reliable and return a microbial community which is similar to a community where DNA is extracted directly from the product. The application of the presented method potentially can be extended to microbial community analysis of liquid environmental samples.

### Conclusion

The discussed filter paper method for microbial community preservation by DNA extraction is an interesting tool to use with fermented foods in the field where other tools are not successful. Besides drying carefully in a clean environment this methods does not require any high-tech or electrical machines. This makes this method very effective for the stabilisation and transport of microbial communities in a nutritious environment like milk.

### Limitations

Resulting bacterial communities show slight variation from bacterial communities constructed using DNA extracted from a liquid sample, but this is not more than variation which can be found between multiple analyses of the same DNA extracts from filter paper disks. Unfortunately, there is no golden standard for DNA stabilisation from fermented milk products to compare with this filter paper method. Another limitation is the risk for contamination. More developments in finding an optimal stabilising method for a longer storage time and a complete DNA extraction method could be done. The biggest risk arises when the papers are not dry enough. The filter paper disks do not contain any preservatives to prevent spoilage, so when the paper remains moist it could form a growth environment for both the organisms present in the sample as well as any contaminants. Regions with a dry climate, like the region in Zambia where the current study is performed, is ideal for this application, while more moist climates could cause difficulties in obtaining the right dryness.

## Additional files

**Additional file 1: Supplement A.** Full DNA extraction protocol used for both filter paper and liquid samples. **Supplement B.** Analysis of community structure using clone libraries. **Supplement C.** Analysis of 16S rRNA amplicon sequencing data for bacterial community reconstruction.

**Additional file 2: Figure S1.** Bacterial composition of liquid and paper samples all originating from the same Mabisi sample. Different colours indicate different species which could originate from different reads. Numbers 1 and 2 indicate the two technical replicates of DNA extraction.

## Abbreviation

OTU: operational taxonomic unit
