# Supplementary material for: Robust sampling and preservation of DNA for microbial community profiling in field experiments
Source: BMC Res Notes. 2019 Mar 22;12:159. doi: 10.1186/s13104-019-4187-2 (PMC6429817; doi:10.1186/s13104-019-4187-2)
Supplement: Supplementary file 1 — Additional file 1: Supplement A. Full DNA extraction protocol used for both filter paper and liquid samples. Supplement B. Analysis of community structure using clone libraries. Supplement C. Analysis of 16S rRNA amplicon sequencing data for bacterial community reconstruction. [file 13104_2019_4187_MOESM1_ESM.docx]

**Additional file to the methods section**

**Supplement A**

**Full DNA extraction protocol used for both filter paper and liquid samples.**

For DNA extraction from liquid samples, 1 mL of fermented milk was spun down (2 minutes, 12000 RPM), after which the supernatant was removed. The cells were re-suspended in a mix of 64 µL EDTA (0.5 M), 160 µL Nucleic Lysis Solution (Promega, Madison, United States), 5 µL RNAse (100 mg/ml), 120 µL lysozyme (10 mg/ml) and 40 µL pronase E (20 mg/ml). Samples were incubated for 60 minutes at 37°C while being shaken at 350 RPM.

For the extraction of DNA from the filter paper discs, a piece, 2 by 2 centimetres, was cut from the middle of the filter paper with a sterile pair of scissors. This piece was positioned at the bottom of a 1,5 mL eppendorf tube with a pair of tweezers. The cells were thoroughly suspended in a mix of 132 µl 0.5M EDTA, 320 µl Nuclei Lysis Solution, 1 µl RNase, 240 µl lysozyme and 80 µl pronase E. Samples were incubated for 24 hours at 37 °C and agitation of 350 RPM.

After the incubation step described above of both liquid samples and paper samples, a standard DNA extraction protocol was used, as follows. 400 µL ice-cold ammonium acetate (5 M) was added and the mixture was cooled on ice for 15 minutes. The mixture was spun down and 750 µL of supernatant was transferred to a tube containing 750 µL phenol. This tube was vortexed and its content spun down (2 minutes, 12000 RPM) and 500 µL of supernatant was transferred to a tube containing 500 µL chloroform. This tube was vortexed and its content spun down (2 minutes, 12000 RPM) and 400 µL of supernatant was transferred to a tube containing 1 ml 100% ethanol and 40 µL sodium acetate (3 M). This tube containing DNA was left to precipitate at -20 °C overnight. The next day, the tube was spun for 20 minutes at 12 000 RPM at 4 °C. The supernatant was carefully aspirated and the DNA pellet was washed by adding 1 mL 70% ethanol. The tube was spun for 10 minutes at 12 000 RPM at 4 °C, after which the supernatant was aspirated again. The DNA pellet was left to dry at room temperature and dissolved in 20 µL 10 mM Tris pH 7.5. DNA concentration were measured using Nanodrop 2000/2000c UV-VIS (ThermoFisher, Waltham, US).

**Supplement B**

**Analysis of community structure using clone libraries.**

For the validation in the laboratory prior to the larger scale field validation experiments, community profiles were analysed using clone libraries. DNA extracts were amplified in the 16S region with a PCR with 16S primers 27F-1492R (Biolegio, Nijmegen, Netherlands) and the following amplification conditions: 94 °C initial denaturation, 10 min; then 34 cycles of 94 °C, 30 sec; 55 °C, 30 sec; 72 °C, 1 min, followed by a 72 °C final extension, 10 min. PCR products were cleaned using a PCR clean-up kit (Macherey-Nagel, Düren, Germany) according to the manufacturer’s instructions. Clone libraries were constructed using the StrataClone PCR Cloning Kit (Stratagene, San Diego, California, United States) according for the manufacturer’s instructions. Resulting clone libraries were sequenced using Sanger sequencing (Eurofins Scientific, Brussels, Belgium) with T3 primer. Sequence data were compared to the NCBI database using BLAST (1). Matches from the blast search were used to construct the bacterial community. Results are in Figure S1.

**Supplement C**

**Analysis of 16S rRNA amplicon sequencing data for bacterial community reconstruction.**

The extract containing DNA from all organisms in the community was sent for bacterial 16S rRNA gene amplicon paired-end sequencing of the V4 hypervariable region (341F-785R) on the MiSeq Illumina platform by LGC genomics (Berlin, Germany). For further data processing and statistics the QIIME pipeline (2), modified from Bik et al (3) was used as follows. Paired-end reads were joined using join_paired_ends.py (with minimum overlap 10 basepairs) after which sequences were trimmed and filtered using cutadapt (v1.11 -q 20, -m 400, Martin 2011) using the known primer sequences CCTACGGGNGGCWGCAG and GACTACHVGGGTATCTAAKCC to trimmed both sides of the sequence. These trimmed sequences were then checked for chimera’s, using uchime (v4.2.20, gold database, Edgar et al 2014), and sequences with a chimera score lower than 0.28 were retained. After the trimming and filtering steps sequences were clustered (UPGMA) into operational taxonomic units (OTUs) after quality check using pick_open_reference_otus.py (-s 0.1, -enable_rev_strand_match TRUE, -align_seqs_min_length 75, -pick_OTU_similatiry 0.95). Taxonomy of the resulting OTUs was assigned to representative sequences using the Greengenes (v13.5) rRNA database. This algorithm gives a representative sequence for an OTU, which were used to perform a local blast using the gold database from uchime. The taxonomy from the top BLAST hit was used for further data processing.

1. Altschul SF, Gish W, Miller W, Myers EW, Lipmanl DJ. Basic Local Alignment Search Tool. J Mol Biol. 1990;215:403–10.

2. Caporaso JG, Fierer N, Peña AG, Goodrich JK, Gordon JI, Huttley GA, et al. QIIME allows analysis of high-throughput community sequencing data. Nat Methods. 2010;7(5):335–6.

3. Bik HM, Maritz JM, Luong A, Shin H, Dominguez-Bello MG, Carlton JM. Microbial Community Patterns Associated with Automated Teller Machine Keypads in New York City. mSphere. 2016;1(6):e00226-16.
