# Supplementary figures and images for: Robust sampling and preservation of DNA for microbial community profiling in field experiments
Source: BMC Res Notes. 2019 Mar 22;12:159. doi: 10.1186/s13104-019-4187-2 (PMC6429817; doi:10.1186/s13104-019-4187-2)

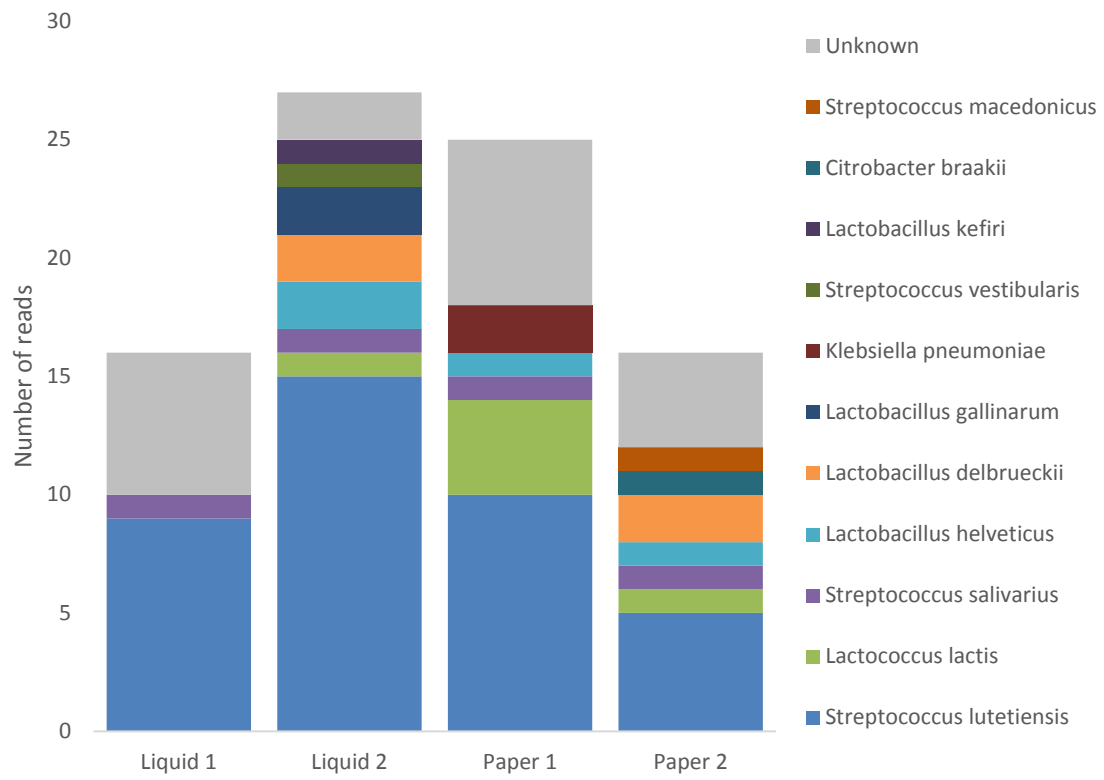

Supplement: Supplementary file 2 — Additional file 2: Figure S1. Bacterial composition of liquid and paper samples all originating from the same Mabisi sample. Different colours indicate different species which could originate from different reads. Numbers 1 and 2 indicate the two technical replicates of DNA extraction. [file 13104_2019_4187_MOESM2_ESM.pdf]
